# Selective feticide in dichorionic diamniotic (DCDA) twins complicated with previable premature rupture of membrane before 24 weeks may be a safe therapeutic alternative to ongoing pregnancy

**DOI:** 10.1186/s12884-024-06361-x

**Published:** 2024-02-26

**Authors:** Caixia Zhu, Haiyan Liu, Hui Zhu, Linhuan Huang

**Affiliations:** Department of Obstetrics and Gynecology, The First Affiliated Hospital of Sun Yat-sen University, Guangzhou, China

**Keywords:** Previable premature rupture of membrane, Selective feticide, Neonatal outcome, Twin pregnancy

## Abstract

**Background:**

To date, there are no clinical guidelines for dichorionic diamniotic (DCDA) twins complicated with previable premature rupture of membrane (PV-ROM) before 24 weeks of gestation. The typical management options including expectant management and/or pregnant termination, induce the risks of fetal mortality and morbidity.

**Objective:**

To explore the feasibility selective feticide in DCDA twins complicated with PV-ROM.

**Study design:**

A Retrospective cohort study, enrolling 28 DCDA twins suffering from PV-ROM in a tertiary medical center from Jan 01 2012 to Jan 01 2022. The obstetric outcome was compared between selective feticide group and expectant management group.

**Results:**

There were 12 cases managed expectantly and 16 underwent selective feticide. More cases suffered from oligohydramnios in expectant management group compared to selective feticide group (*P* = 0.008). Among 13 cases with ROM of upper sac, the mean gestational age at delivery was (33.9 ± 4.9) weeks in the selective feticide group, which was significantly higher than that in the expectant management (*P* = 0.038). Five fetuses (83.3%) with selective feticide delivered after 32 weeks, whereas only one (14.3%) case in expectant management group (*P* = 0.029). However, in the subgroup with ROM of lower sac, no significant difference of the mean gestation age at delivery between groups and none of cases delivered after 32 weeks.

**Conclusion:**

There was a trend towards an increase in latency interval in DCDA twins with PV-ROM following selective feticide, compared to that with expectant management. Furthermore, selective feticide in cases with PV-ROM of upper sac has a favorable outcome.

## Introduction

Previable premature rupture of membrane (PV-ROM), a rare complication occurs before 24 weeks of gestation, has a high risk of neonatal mortality and morbidity [1]. The incidence of PV-ROM is 0.1–0.37% of pregnant women and it’s more frequent in twin than that in singleton pregnancy [2]. Previous studies demonstrated that PV-ROM was correlated with severe perinatal and neonatal morbidity and mortality such as intrauterine infection, stillbirth, early preterm birth, and complications owing to prematurity. The earlier that PV-ROM occurs in dichorionic diamniotic (DCDA) twins, the higher the risk of preterm delivery and consequently poorer the prognosis for neonatal survival. Based on the singleton gestation, the general management options of DCDA twins with PV-ROM include pregnancy termination or expectant management under antibiotic coverage. Myrick and co-workers [3] present that the median interval from RV-ROM to delivery was 13 days in 30 twins under expectant management. Among 60 fetuses, only 16 fetuses survive. It’s reasonable to assume that expectant management for DCDA twins with PV-ROM gave a short latency period. Furthermore, Lin et al [4] reported that the mean latency was 39.6 days in 22 DCDA twins with PV-ROM following expectant management. Wong [5] showed that the median latency of 11 days was reached in cases with PV-ROM expectantly managed and only 43% neonates survived.

In view of the poor outcomes of singleton pregnancies with PV-ROM managed expectantly as well as twin pregnancies, selective feticide was reported alternative in these cases. In 2008, Keselman [6] reported 2 DCDA twins with PV-ROM of upper sac took selective feticide and both cases delivered after 36 weeks. To date, the literature regarding impact of selective feticide on DCDA twins with PV-ROM is limited as more studies focused on expectant management. Small series present a favorable outcome for DCDA twins with PV-ROM following selective feticide [7–9]. Zajicek [10] showed that all of 6 cases following selective feticide survived and the median latency was 129 days, suggesting a possible benefit trend for DCDA twins with PV-ROM. Our hospital has offered this procedure for the past 8 years for DCDA twins with PV-ROM when the patients chooses so after careful consulting. The purpose of this study was determining the obstetric outcomes of DCDA twins complicated with PV-ROM managed expectantly versus selective feticide. In addition, we wished to find out the latency interval for cases following selective feticide.

## Materials and methods

### Study design

This was a retrospective cohort study of all DCDA twins complicated with rupture of membranes (ROM) between 13 and 24 weeks of gestation, which managed in a terity medical center between Jan 01, 2014 and Jun 01, 2022. This study was approved by The First Affiliated Hospital, Sun Yat-sen University and the informed consent was waived. Diagnosis of ROM was confirmed based on the evaluation of continuous leakage of clear amniotic fluid on speculum examination and oligohydramnios by ultrasound. Chronicity was demonstrated by ultrasound finding with placental pathology when delivery. Gestational age was determined either by a combination of last menstrual period date and early ultrasound finding or information of assisted reproduction.

All cases received a detailed consultation regarding the management regimen including termination of pregnancy, expectant management, and selective feticide. The cases underwent termination of pregnancy once diagnosis of ROM was excluded. In the expectant management group, careful monitoring was performed for the mother and fetus. First, general condition was monitored every day, including body temperature, pulse, uterine pressure, and vaginal secretion. The laboratory examination including white blood cell count, C-reactive protein, procalcitonin (PCT), and secretion culture, was regularly taken for all cases. In addition, fetal growth and amniotic fluid volume was monitored regularly. In the selective feticide group, all cases underwent selective feticide within a week following the diagnosis of ROM. There was not any differences in parameters, including white blood cell counts, C-reactive protein (CRP), and procalcitonin, between the expectant and feticide groups. The antibiotic therapy was applied for all cases once they were diagnosed as PPROM. Meanwhile, blood test, GBS screening, vaginal and cervical swab were performed at admission for PPROM. For the manage expectant group, the regimen of amoxicillin-containing antibiotics was taken for 5 days. The same antibiotics regimen was applied for the cases after the selective feticide for 2 days.

Once ROM was diagnosed, the initial assessment for maternal and fetal status was performed for both two groups. Induced abortion should be recommended in the cases of contraindications, such as infection (chorioamnionitis, fetal distress, placental abruption or all conditions that warrant immediate termination.

All data extracted from obstetric and neonatal record of cases following expectant management or selective feticide. The primary outcomes were compared between expectant management group and selective feticide group. Then subgroup analysis was performed based on the location of sac rupture (A or B).

### Outcomes

Primary outcomes rate of delivery after 32 weeks of gestation. Secondary outcomes included gestational age at delivery, latency interval between ROM and delivery, rate of intrauterine infection, live birth, and neonatal complication.

### Statistical analysis

Data were analyzed using SPSS (IBM SPSS Statistics 24 Armonk, NY). Categorical variables were compared using Chi-square test or Fisher exact t test. Continuous variables were compared using student’s t test. *P* vale ≤ 0.05 was considered as statistically significant.

## Results

**Fig. 1:**
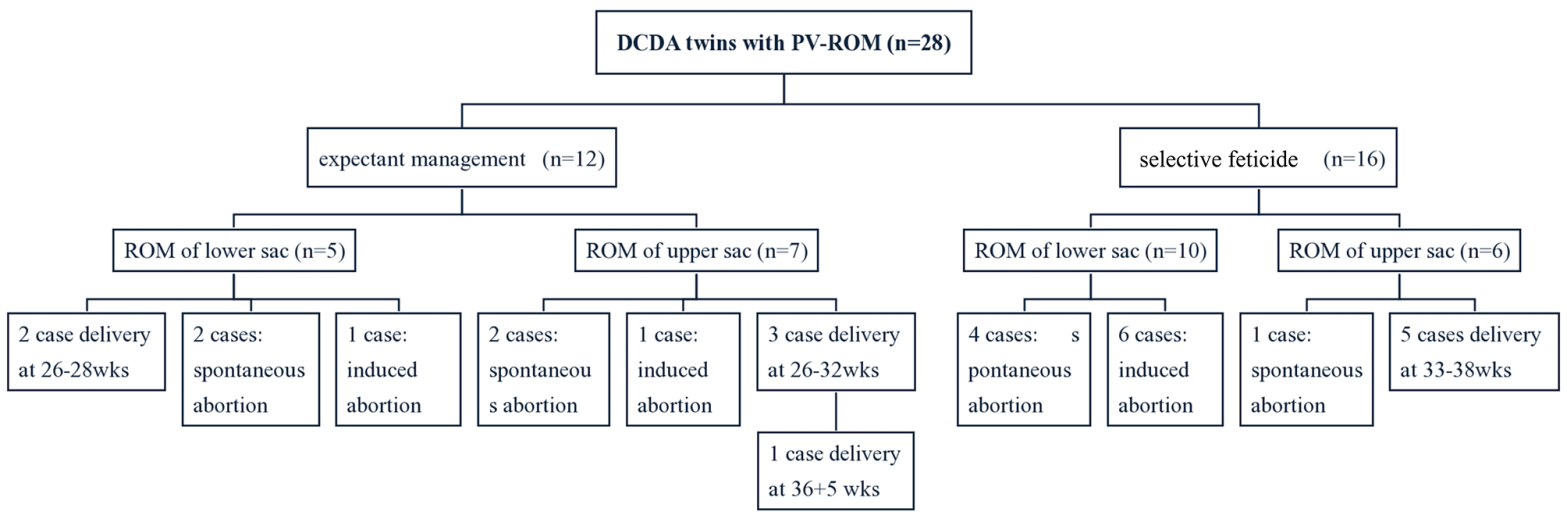
Obstetric outcome of DCDA twins with PV-ROM managed expectantly or selective feticide

A total 28 cases of dichorionic diamniotic twin gestation met the inclusion criteria. There were 16 cases chose selective feticide and 12 cases chose expectant management (Fig. 1). The demographic characteristics were outlined in Table 1. No significant difference in the mean maternal age, parity, the risk of artificial reproductive technique, mean gestational age at PV-ROM and location of ROM between two groups.

**Table 1:** Demographic characteristics of twins pregnancies with PV-ROM^a^

	expectant management (*N* = 12)	selective feticide (*N* = 16)	*P* value
maternal age, years	33.2 ± 4.5	31.8 ± 3.6	0.382
AMA^b^	5 (41.7%)	3 (18.8%)	0.231
nulliparous	4 (33.3%)	2 (12.5%)	0.354
ART^c^	9 (75.0%)	12 (75.0%)	1.000
mean GA^d^ at PV-ROM (wks)	20.1 ± 2.9	19.4 ± 2.5	0.509
location of PV-ROM			
upper sac	7 (58.3%)	6 (37.5%)	0.274
lower sac	5 (41.7%)	10 (62.5%)	

^a^PV-ROM: previable premature prelabor rupture of membrane

^b^AMA: advanced maternal age

^c^ART: assisted reproduction technique

^d^GA: gestational age

Table 2 summarized the obstetric outcomes between two groups. Compared to expectant management, less cases suffered from intrauterine infection in selective feticide group. In expectant management group, only one case delivered after 32 weeks of gestation, while more cases (31.3%) delivered after 32 weeks of gestation in the selective feticide group. More cases suffered from oligohydramnios in expectant management group compared to selective feticide group (*P* = 0.008).

**Table 2:** Obstetric outcomes of twins pregnancies with PV-ROM^a^

	expectant management (*N* = 12)	selective feticide(*N* = 16)	*P* value
intrauterine infection	8 (66.7%)	8 (50.0%)	0.378
oligoamnios	6 (50.0%)	1 (6.3%)	0.008
survival rate	6 (50.0%)	5 (31.3%)	0.315
mean GA^b^ at delivery	24.9 ± 5.5	25.9 ± 7.2	0.701
delivery ≥ 32wks	1 (8.3%)	5 (31.3%)	0.196
layency interval (IQR)	38.6 (5-139)	52.2 (7-158)	0.452

^a^PV-ROM: previable premature prelabor rupture of membrane

^b^GA: gestational age

**Fig. 2:**
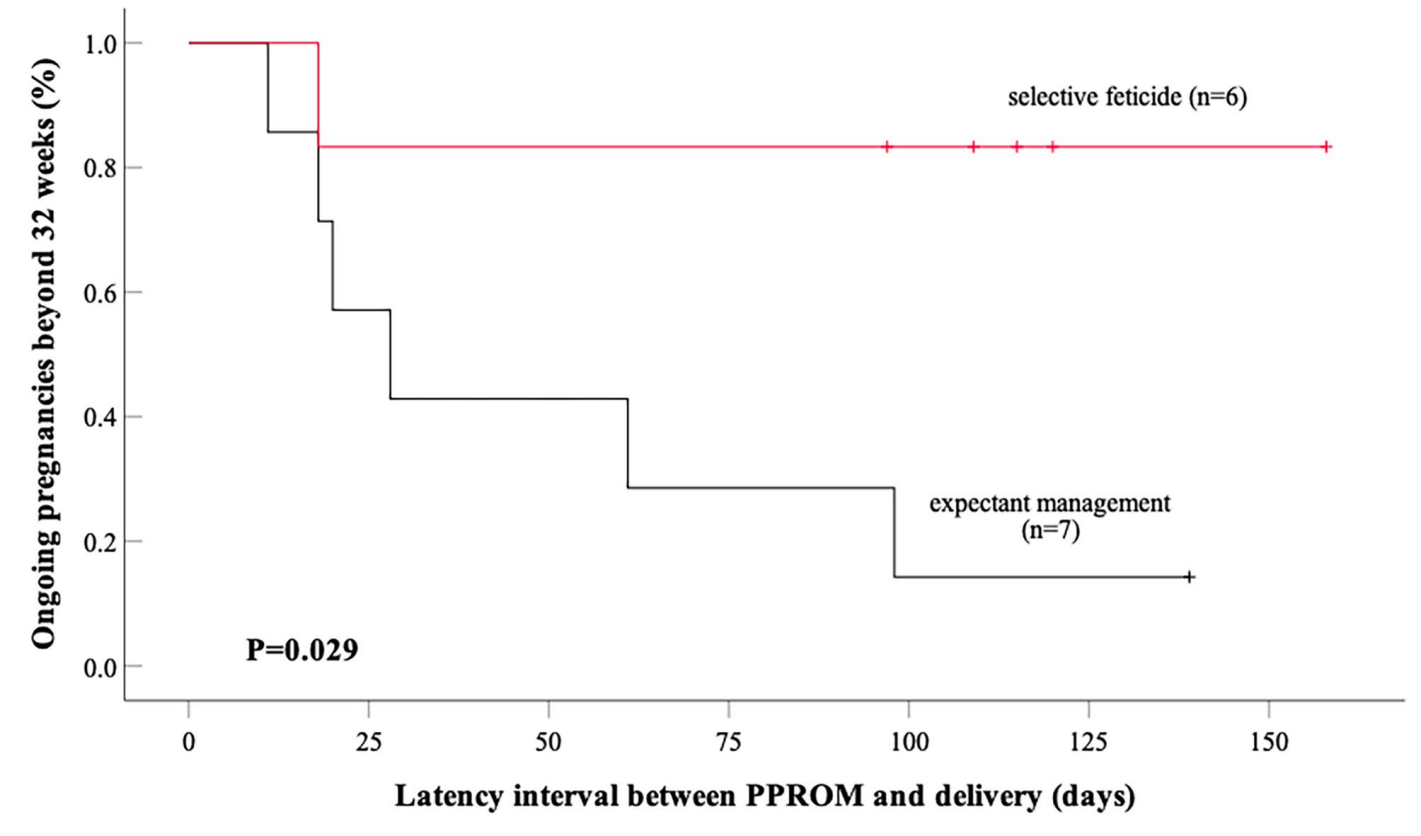
Proportion of ongoing pregnancies beyond 32 + 0 weeks in twin pregnancies with PV-ROM of upper sac

The obstetric outcomes of subgroups were present Table 3. Among 13 twin pregnancies with ROM of upper sac, 9 cases survived. The mean gestational age at delivery was 33.9 ± 4.9 weeks in the selective feticide group, which was significantly higher than that in the expectant management (26.6 ± 6.2, *P* = 0.038). The interval time between diagnosis of ROM and delivery in selective feticide group was 52.2 ± 50.3 days, longer than that in expectant group (38.6 ± 41.0 days). As shown in Fig. 2, five cases (83.3%) with selective feticide delivered after 32wks, whereas only one (14.3%) case in expectant management group (*P* = 0.029).

**Table 3:** Obstetric outcomes of twins pregnancies with PV-ROM^a^ in different subgroup

	Upper sac		*P* value	Lower sac		*P* value
	expectant management (*N* = 7)	selective feticide (*N* = 6)		expectant management (*N* = 5)	selective feticide (*N* = 10)	
intrauterine infection	5 (71.4%)	1 (16.7%)	0.103	3 (60.0%)	7 (70.0%)	1.000
survival rate	4 (57.1%)	5(50.0%)	0.559	2(40.0%)	0(0%)	0.095
mean GA^b^ at delivery	26.6 ± 6.2	33.9 ± 4.9	0.038	22.6 ± 3.9	21.2 ± 2.5	0.471
delivery ≥ 32wks	1 (14.3%)	5 (83.3%)	0.029	0 (0%)	0 (0%)	1.000

^a^PV-ROM: previable premature prelabor rupture of membrane

^b^GA: gestational age

There was a possible increased trend for risk of intrauterine infection in the expectant management group compared to those in the selective feticide (71.4% vs. 16.7%, *P* = 0.103). None of the fetuses of cases with ROM of the lower sac was born after 32 weeks of gestation. The mean gestational age at delivery was similar between two groups, which is less than 24 weeks of gestation. There was no significant difference of intrauterine infection rate, survival rate and mean gestational age between these two groups.

Table 4 present the neonatal outcomes of all survival fetuses (*n* = 11). There were 2 neonatal death in expectant management group. The birth weight was lower in newborns of expectant management group, while less cases with the neonatal complications were observed in selective feticide group.

**Table 4:** Neonatal outcomes of survival twins with PV-ROM^a^

case no.	GA^b^ at ROM (twin A/B)	management	GA at delivery (weeks)	mode of delivery	birth weight (gram)	neonatal complication
1	22 + 6(A)	expectant	26 + 6	VD^c^	A:900 B:960	RDS^e^, PDA^f^, sepsis, ICH^g^ RDS, PDA, sepsis
2	22 + 3 (B)	expectant	26 + 1	CS^d^	A:690 B:9330	death RDS
3	16 + 6 (B)	expectant	36 + 5	CS	A:2550 B:2370	normal normal
4	21 + 3 (B)	selective feticide	38	VD	2800	normal
5	23 + 2 (B)	selective feticide	36 + 6	VD	2800	normal
6	21(B)	selective feticide	37 + 2	VD	1440	normal
7	17 + 4 (B)	selective feticide	33 + 6	VD	1880	PDA
8	15 + 4 (B)	selective feticide	33 + 3	VD	1670	normal
9	23 + 5 (B)	expectant	31 + 4	CS	A:1460 B:1680	death RDS, PDA sepsis
10	23 + 1 (A)	expectant	26 + 2	VD	A:940 B:1000	RDS, PDA sepsis, ICH RDS
11	15 + 3 (B)	expectant	29 + 2	CS	A:990 B:1330	RDS PDA spesis RDS

^a^PV-ROM: previable premature prelabor rupture of membrane

^b^GA: gestational age

^c^VD: vaginal delivery

^d^CS: cesarean section

^e^RDS: respiratory distress syndrome

^f^PDA: patent ductus arteriosus

^g^ICH: intracranial hemorrhage

## Discussion

In present study, we present cases with selective feticide in DCDA twins with PV-PROM of upper sac having favorable outcomes, including low risk of oligohydramnios. Compared with the expectant management group, more cases attaining at least 32 weeks in the selective feticide group. However, there was no significantly difference in the obstetric outcomes in DCDA twins with PV-PROM of lower sac whether selective feticide was performed or not.

Zajicek and colleagues [11] reported that 13 cases of PV-PROM following selective feticide delivered at 39 weeks, which was greater than that 30 weeks of gestation in cases underwent expectantly management. The cases complicated with intrauterine infection were excluded, resulting in the median gestational age at delivery in above study was substantially longer than our study. The improvement of neonatal care might indicate the higher survival rate. Furthermore, in another study, 6 DCDA twins with PV-ROM following selective feticide delivered at 33–40 weeks, while 3 patients delivered before 32 weeks with expectant management [12]. It’s seeming that selective feticide has an advantage over expectant management. The interval time between ROM and delivery in selective feticide group was longer than that in expectant management group, but not reach significant, probable due to the small number of cases. It might be explained by a cessation of amniotic fluid leakage after selective fetocide. Another possible explanation would be protective effect of the cervical mucous plug.

For all cases from PV-PROM of upper sac, the gestational age at delivery reaching 33.9 ± 4.9 weeks was far greater in the selective feticide group compared with 26.6 ± 6.2 weeks in expectant management group. In addition, a lower rate of preterm delivery before 32 weeks of 16.7% in selective feticide group compared to 85.7% in the expectant management group. It’s potential that an intact lower sac might protect the fetuses with ROM of upper sac. Previous study showed that the incidence of intrauterine infection was 15% and all in the cases with expectant management. However, in present study, the risk of intrauterine infection was up to 71.4% in expectant management group compared to 16.7% in the selective feticide group.

Nevertheless, selective feticide for cases with PV-ROM of lower sac showed no benefit compared with expectant management. No cases delivered after 32 weeks of gestation. It’s still conventual to perform selective feticide for the DCDA twins of PV-PROM. Most of literature focused on the twins with PV-ROM was small series. Dinsmoor [8] observed that the survival rate was only 30% in 10 twins with PV-ROM following expectantly managed. In a retrospective cohort which included 27 DCDA twins with PV-ROM following expectant management, Wagner [12] reported 60% of cases delivered after 24 weeks and the survival rate of fetus without major complications were 40%. De Catte and co-workers [7] described that none of nine cases of PV-PROM with expectant management delivered after 30 weeks of gestation. In accordance with previous study, the median gestational age at delivery from expectant management group in present study was 24.9 ± 5.5 weeks.

To the best of our knowledge, no larger study that assesses the optimal management for the DCDA twins with PV-ROM. Our results showed important information that could consulting the DCDA twins with this uncommon condition. Based on our results, selective feticide showed a possible trend for favorable outcomes, especially for the cases with PV-ROM of upper sac. More cases with ROM of lower sac in selective feticide group could possibly impact the results, because the prognosis of impaired lower sac is poor than the prognosis of intact lower sac. The potential risk in prolongation of pregnancy should be considered when we consult with patients.

It’s the first study that reported selective feticide can be offered for DCDA pregnancies with PV-ROM of upper sac. When patients with PV-ROM required maintain pregnancy despite the risk, selective feticide seemed to be associated with an excellent outcome in the neonates owing to prolonged gestational age at delivery. The main limitation of present study is the small number of cases in our cohort, which is due to the rarity of PV-ROM in DCDA twins. A large series should be further studied. Another disadvantage is the retrospective nature of present study which leading to the validity of the data limited by selection bias. Numerous potential confounders, including antibiotic regimen, prior abortion, group B streptococcus status, might influence the obstetric outcomes in this study. When accounting for the reduced fetus after selective feticide, the survival rate of fetus in the expectant management group seems high.

## Conclusion

In conclusion, in DCDA twins complicated with PV-ROM of upper sac, selective feticide seems to be an adequate option leading to favorable obstetric outcomes compared with expectant management, since the gestational age at delivery was higher when patients underwent selective feticide. The chance of survival of both fetuses by expectant management seems to be higher if the ROM occurs in the lower sac but seems to be associated with high risk of neonatal complications. Like the multifetal reduction of high-risk multiple, selective feticide in PV-ROM represents a difficult choice of sacrificing one fetus to benefit the other. Therefore, selective feticide might be an option to be considered for DCDA twins with PV-ROM who wish to maintain pregnancy, especially in cases with ROM of the upper sac. A larger cohort studies of selective feticide for PV-ROM DCDA twins to further explore.

## Data Availability

The datasets generated and/or analysed during the current study are not publicly available due patient privacy but are available from the corresponding author on reasonable request.
